# Successful corneal endothelium preservation in the management of epithelial downgrowth with 5-fluorouracil injections and membranectomy

**DOI:** 10.1186/s12886-024-03719-9

**Published:** 2024-10-15

**Authors:** Ka Wai Kam, Andre Ma, Joanna Ng, Paul Choi, Wilson Wai Kuen Yip, Alvin L. Young

**Affiliations:** 1 Department of Ophthalmology and Visual Sciences, Prince of Wales Hospital, 30 Ngan Shing Street, Shatin, New Territories Hong Kong SAR, China; 2 Department of Ophthalmology and Visual Sciences, The Chinese University of Hong Kong, Shatin, Hong Kong SAR, China; 3 Department of Ophthalmology and Visual Sciences, Alice Ho Miu Ling Nethersole Hospital, Taipo, New Territories Hong Kong SAR, China; 4 Department of Anatomical and Cellular Pathology, The Chinese University of Hong Kong, Shatin, Hong Kong SAR, China

**Keywords:** Epithelial downgrowth, Cataract surgery, 5-Fluorouracil, Mitomycin-C, Methotrexate

## Abstract

A 74-year-old Chinese man underwent extracapsular cataract extraction in his right eye and developed a translucent iris membrane 4 months later. He was treated with two intracameral 5-FU injections and membranectomy at 2 weeks apart. At one year following the second membranectomy, the patient maintained a clear cornea without residual or recurrent membrane, an endothelial cell density of 1072 cell/mm^2^, a visual acuity of 20/50 and a normal intraocular pressure. Our technique of using dispersive and cohesive viscoelastics in protecting the corneal endothelium from intracameral 5-FU, helped preserve corneal endothelial cells and maintain corneal clarity at one year after surgery.

## Introduction

Epithelial downgrowth (ED) is a rare and sight-threatening complication following intraocular surgery. It is characterized by invasion of corneal and/or conjunctival epithelial cells into the intraocular space and proliferation over the iris, iridocorneal angle and corneal endothelium. These may lead to refractory glaucoma by secondary angle closure, corneal decompensation and sight loss [1–6]. In a thirty-year review, Weiner et al. reported 52% of the surgically-treated eyes with ED and all medically-treated patients eventually required enucleation [1]. Radical removal of the invading epithelial cells, by means of cryotherapy, radical excision with or without intraocular lens (IOL) explant, followed by reconstructive surgeries were often considered to be the only hope for cure. The trauma imposed imparts an overall grim visual prognosis and foretells a prolonged rehabilitation process. With the advancements in imaging, laser, and the advent of intracameral chemotherapy, clinicians were now able to detect the disease at an earlier stage, and explore methods that could preserve healthy structures while targeting destruction to the affected parts. Nonetheless, intracameral 5-fluorouracil (5-FU) or mitomycin-C (MMC) are cytotoxic and could lead to corneal decompensation especially in eyes with low endothelial cells. Herein, we report a case of early ED that we managed with repeated intracameral 5-FU and membranectomy with favorable outcome.

## Case report

**Fig. 1:**
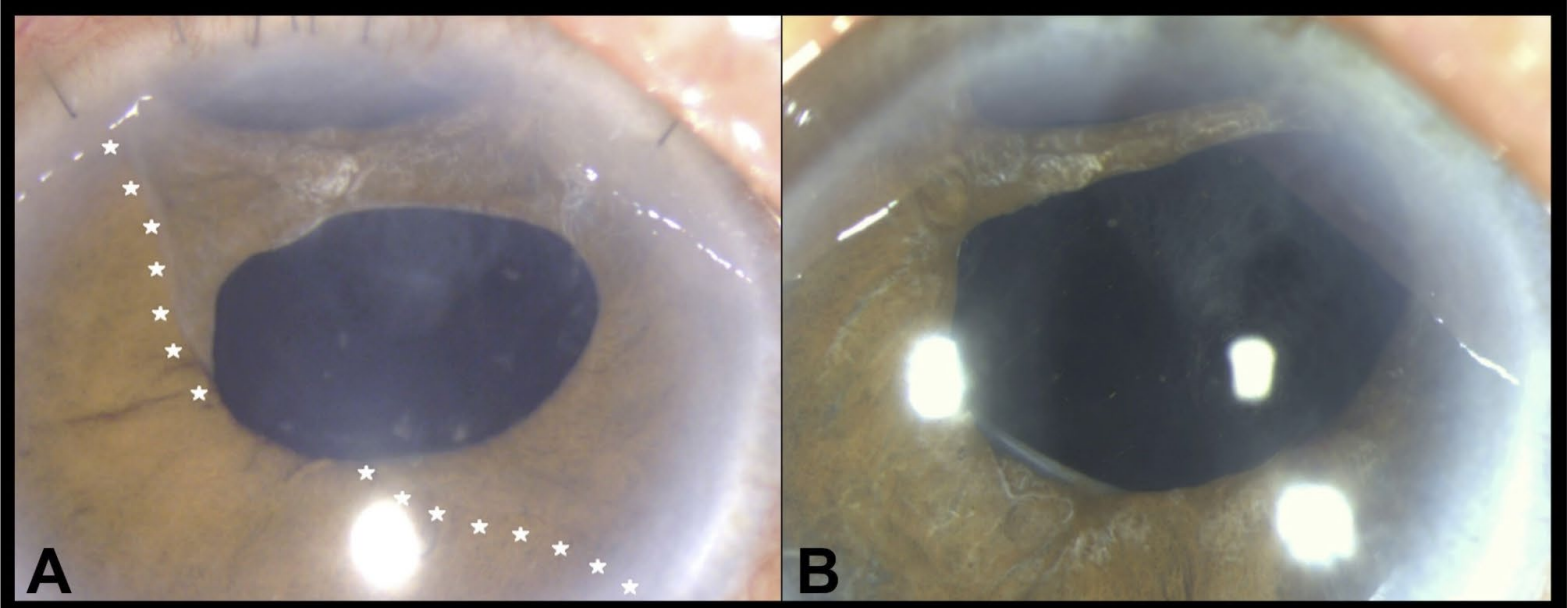
Illustrates the sheet-like epithelial downgrowth in the form of a translucent membrane over the iris (**A**), spanning across from 10 to 5 o’ clock. Superior iridodialysis was noted from 11 to 1 o’ clock. Post-operative, no membrane was seen overlying the anterior surface of the optics and iris (**B**). The white star outlines the advancing border of the iris membrane

**Fig. 2:**
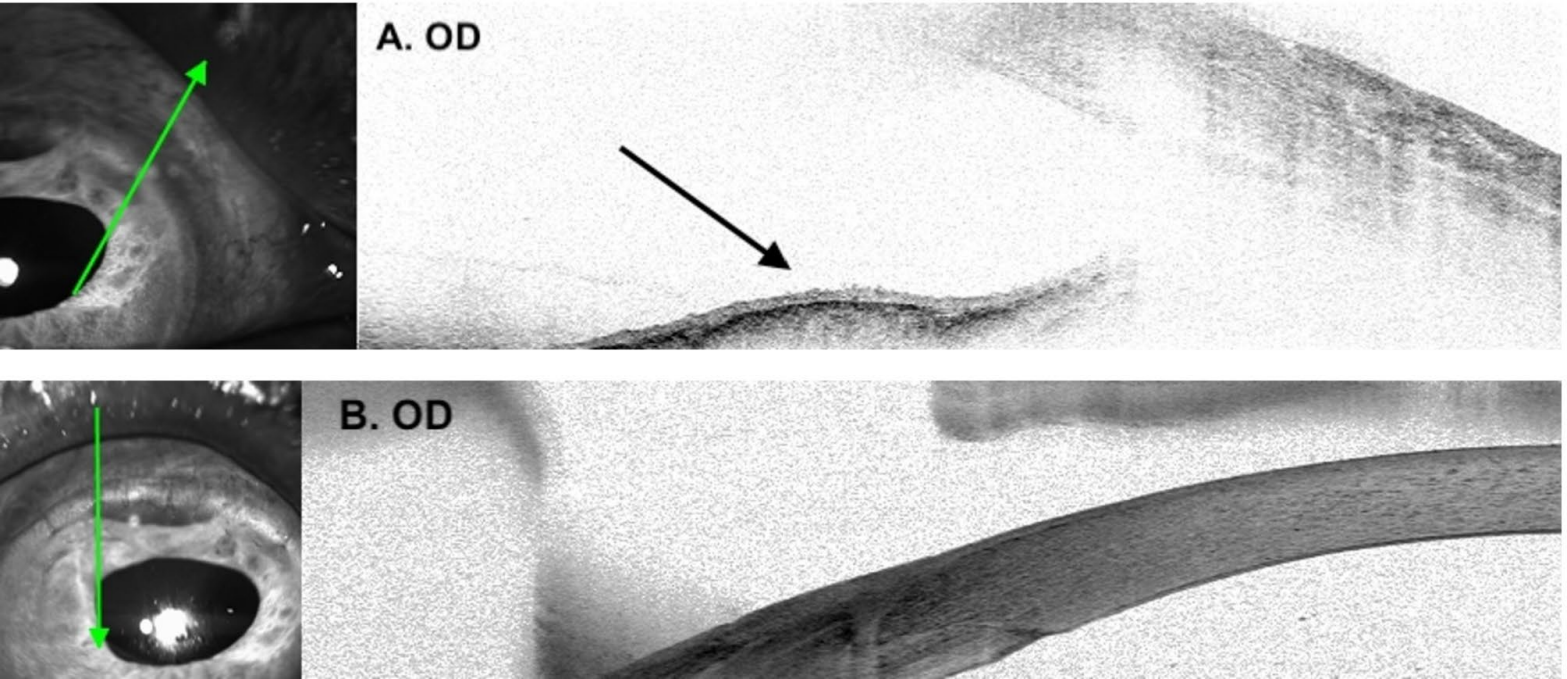
Anterior segment OCT images of the right eye showing presence of a hyperreflective membrane (black arrow) over the anterior iris surface (**A**) with no retrocorneal involvement (**B**)

A 74-year-old Chinese man with a mature cataract underwent planned ECCE with implantation of a rigid Poly(methyl methacrylate) IOL into the posterior chamber. Intraoperatively, a superior iridodialysis from 11 to 1 o’ clock was noted after repeated attempts to reduce the prolapsing iris. At postoperative one-week, wound dehiscence with iris prolapse was noted and during the repair, the surgeon tried to repair the dialysis by applying full thickness transcorneal sutures to fixate the peripheral iris. Four months later, the patient developed mild pain and redness in the right eye. Slit lamp examination revealed trace anterior chamber cells and a translucent membrane on the iris, extending from the superior root and spanning across 10 to 5 o’clock (Fig. 1A). No membrane was seen over the corneal endothelium, anterior chamber angle or the intraocular lens. Diode laser was applied to the iris membrane which blanched exclusively in the area suspicious of ED. Anterior segment optical coherence tomography showed a hyperreflective membrane on the iris surface without retrocorneal involvement (Fig. 2).

**Fig. 3:**
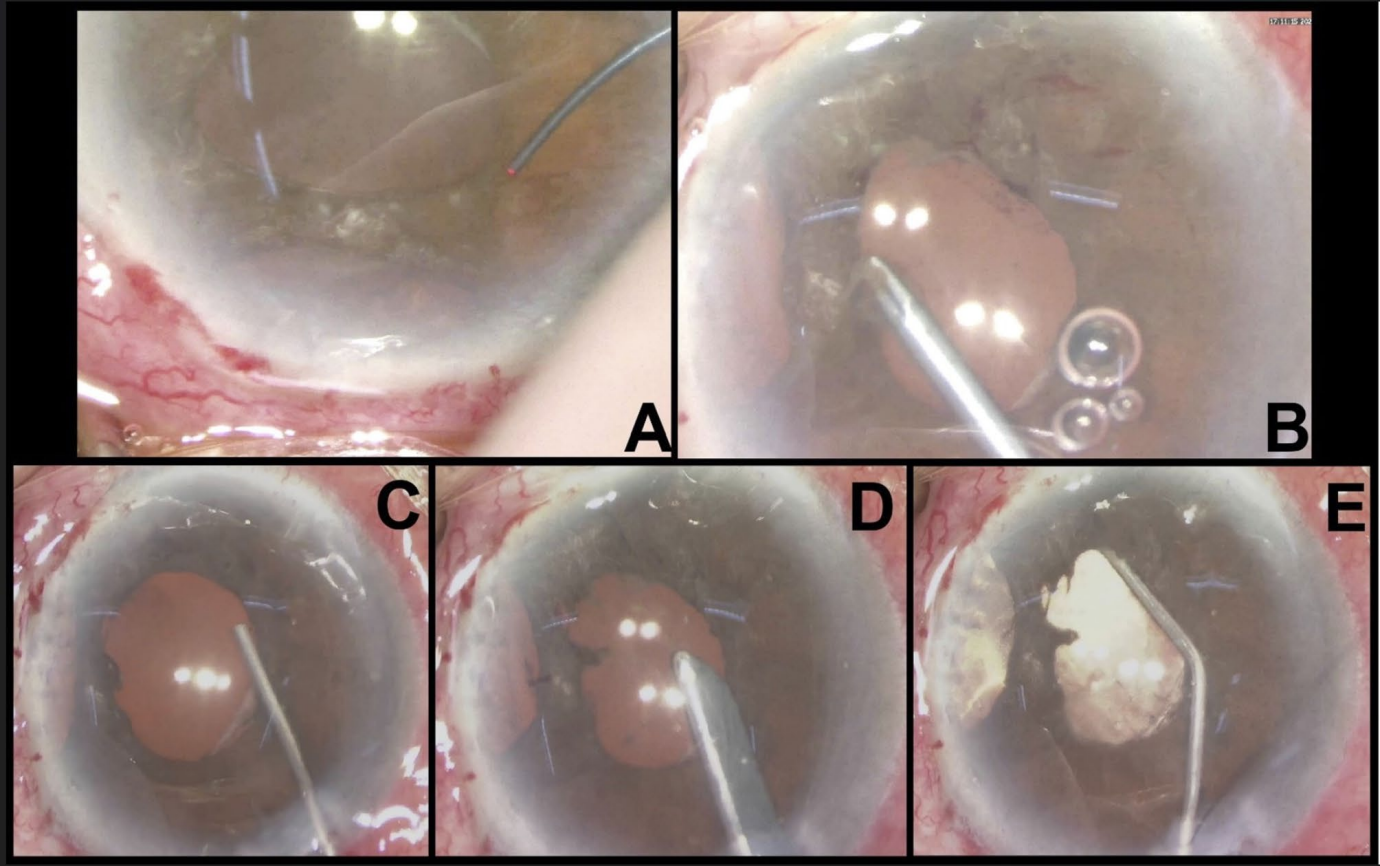
illustrates the key steps of the first surgical procedure involving intracameral 5-FU injection and membranectomy. **A** depicts the application of endolaser to demarcate the iris membrane, epithelial downgrowth blanched while adjacent normal iris tissue charred and darkened after laser application. **B** depicts the peeling of membrane with microforceps. **C** depicts the injection of Viscoat to coat the corneal endothelium followed by ProVisc using a soft-shell technique. **D** depicts the removal of Provisc with Simcoe cannula. **E** depicts the injection of 5-fluorouracil (1.0 mg/mL) intracamerally above and underneath the iris, and to the iridocorneal angle of the involved clock hours

**Fig. 4:**
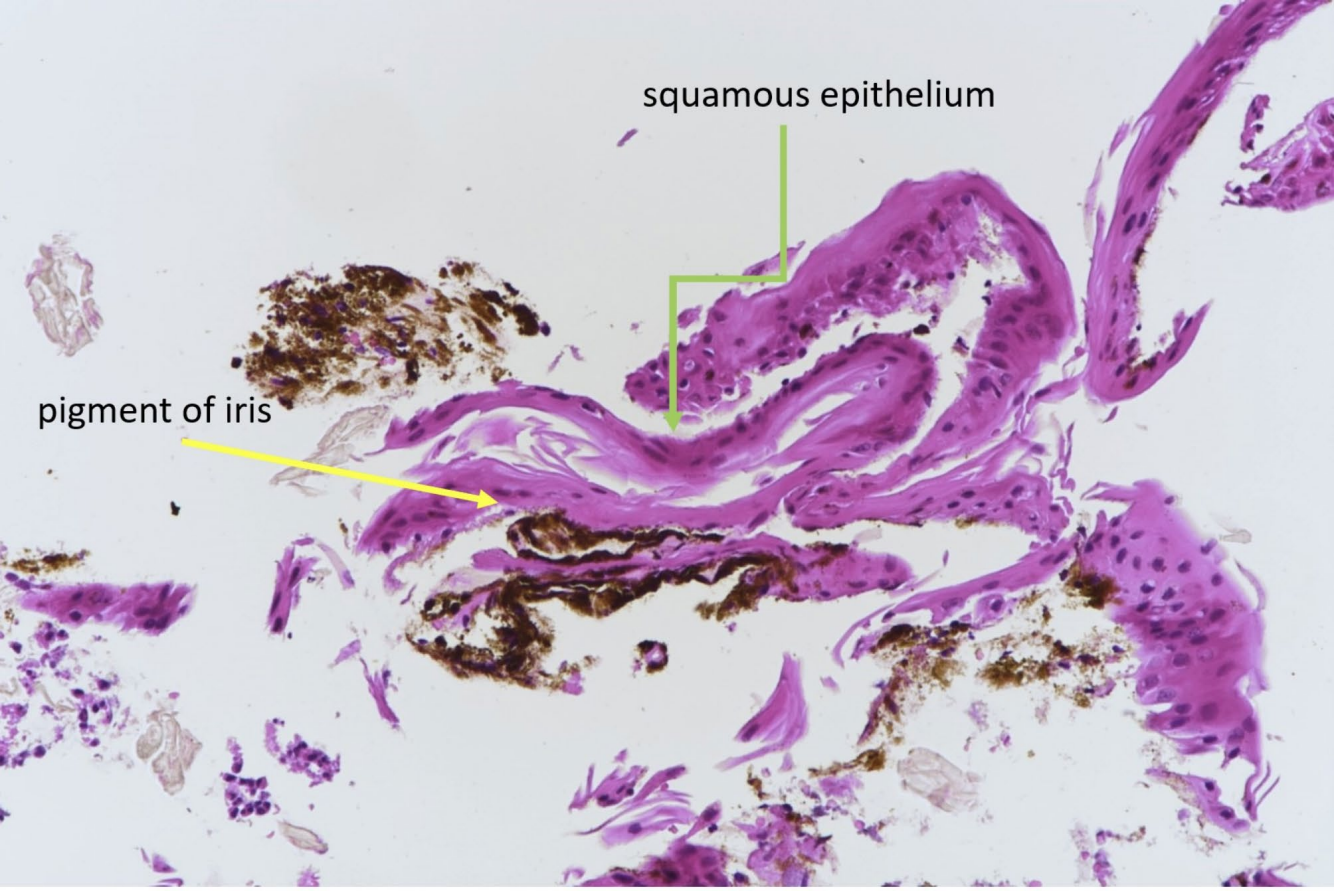
Histopathological photograph at 200X magnification of the stripped membrane showing the presence of iris pigment (yellow arrow) and squamous epithelium above (green arrow) with Hematoxylin & Eosin stain

The patient underwent a two-staged intracameral 5-FU combined with membranectomy (Fig. 3). The operation was performed under retrobulbar anaesthesia and intracameral 5-FU was prepared at a concentration of 1 mg/0.1 ml. Intraoperatively, the superior peritomy was reopened and all the limbal sutures were removed. Scraping of the superior peripheral cornea, Tenon’s capsule and sclera was performed using a Tooke knife followed by thorough irrigation of the ocular surface with balanced salt solution to ensure complete removal of epithelial cells. Endo-laser (Constellation^®^ Vision System, Alcon, United States) was applied to demarcate the extent of the membrane. The iris membrane was then peeled with 23-gauge micro-forceps (23 g MST Micro-Holding forceps and 23 g Ahmed Micro-Graspers, MicroSurgical Technology, United States), A soft-shell technique was applied wherein a layer of dispersive viscoelastic was injected first to protect the corneal endothelium (Viscoat, Alcon), followed by a ball of cohesive viscoelastic (ProVisc, Alcon) to maintain the anterior chamber. After removal of all membranes, the ProVisc was carefully aspirated by Simcoe cannula leaving behind the thick layer of Viscoat. Intracameral 5-FU was slowly infused above and beneath the iris surface using a 1.0 mL syringe and a 30-gauge cannula. A total of 1.6mL of 5-FU at 1 mg/0.1mL (16 mg) was injected and left in-situ. At the end of the surgery, we prescribed prophylactic oral acetazolamide to prevent postoperative IOP spike. The same procedure was repeated 2 weeks after, with an additional 1.0mL of 5-FU at 1 mg/0.1mL injected (10 mg). As no recurrent membrane was noted, only 5-FU injection was performed at the second operation. Histopathologic analysis demonstrated squamous epithelium and confirmed the diagnosis of ED (Fig. 4). At one year after the second operation, the patient remained pain-free and asymptomatic. Examination revealed a quiescent eye with no recurrent or residual membrane (Fig. 1B) and a best-corrected distance VA of 20/50. The cornea endothelial cell density was maintained at 1072 cell/mm^2^ on specular microscopy with a normal IOP.

## Discussion

We presented a patient with membranous ED after ECCE that was managed with combined intracameral 5-FU and membranectomy. In view of the scarce reports of both ED and intraocular chemotherapy, we summarized the available evidence.

### 5-fluorouracil (5-FU)

As a pyrimidine analogue, intracameral 5-FU is an effective treatment for ED by inhibiting proliferating epithelial cells [7–12]. Dosages between 0.04 and 1 mg in single or sequential injections were reported [9, 10]. Animal studies suggested that the threshold concentration for corneal endothelial toxicity lied between 0.1 and 1 mg/0.1ml. [13] Wong et al. reported a case of ED following DSAEK with a nearly identical endothelial cell count at one year after intracameral 5-FU at 0.04 mg/0.1ml [10].

Sequential injections were more often reported as a single dose is allegedly insufficient. 5-FU only targets proliferating cells and hence cells initially in the rest phase may generate a recurrence after a single injection. Sequential treatment therefore helps maximize the clearance of epithelial cells [7, 10]. In most reports of sequential 5-FU injections, the treatment interval was set between 2 and 3 weeks.

5-FU monotherapy achieved an average success rate of 58% among 12 reported cases (see Table 1). On the other hand, five case reports described a combined intracameral 5-FU, at a dosage ranging from 0.25 to 1 mg/0.1 ml, with membranectomy that arrested the disease without progression or recurrence in all cases. We speculate that the mechanical membranectomy had drastically reduced the number of proliferating epithelial cells rendering the 5-FU more effective. However, the small number of cases forbade meaningful statistical comparisons between groups. The existing evidence also suggested that combined treatment could achieve fewer recurrence for a longer period post-operatively. However, the data should be interpreted with the knowledge that the combined group were also treated earlier than the monotherapy group. (combined = 12.1 ± 12.0 months VS. monotherapy = 15.6 ± 19.4 months, *P* = 0.9124). Like our patient, who was diagnosed at 4 months after the incident surgery, these ED were likely more localized and amenable to interventions, and therefore a better long-term outcome.

**Table 1:** Reported cases of ED treated with intracameral 5-FU, combined 5-FU with membranectomy, methotrexate and mitomycin-C

	Age	Sex	Incident surgery	Time from incident surgery or recurrence (m)	Main features	Concentrations	No. of injection	Recurrence	Recurrence-free /follow-up period (m)	Corneal Decompensation following treatment
**5-FU monotherapy**										
1	65	M	DSAEK [9]	14	Retrocorneal membrane	1 mg/0.1 ml	2	Yes	3	Yes + PKP at 3 m
2	69	M	Phaco + IOL [9]	18	Retrocorneal + angle membrane	1 mg/0.1 ml	2	-	5	Yes + DSAEK at 4 m
3	79	F	DSAEK [10]	6	Retrocorneal membrane	0.4 mg/0.1 ml	1	-	6	No
4	70	F	PKP [17]	72	Retrocorneal membrane	0.2 mg/0.1 ml	2	-	14	Yes + PKP at 5 m
5	44	M	PKP [7]	10	Retrocorneal + iris membrane	0.5 mg/0.1 ml	2	-	5	No
6	73	M	PKP [16]	2	Retrocorneal pigmented line	1 mg/0.1 ml	2	-	9	Yes, at 1 m, await graft
7	58	M	PKP [20]	3	Retrocorneal membrane	0.5 mg/0.1 ml	2	-	5	Yes, at 5 m, await graft
8	73	F	DMEK [3]	14	Retrocorneal pearl	0.2 mg/0.1 ml	1	-	48	No
9	65	M	DSAEK [18]	6	Retrocorneal membrane	0.5 mg/0.1 ml	3	Yes	2	Yes + PKP at 5 m
10	69	M	DSAEK [18]	30	Retrocorneal membrane	0.5 mg/0.1 ml	2	Yes	2	No
11	74	M	Phaco + IOL [19]	6	Retrocorneal membrane	0.5 mg/0.1 ml	3	Yes	16	No
12	77	F	Phaco + IOL [21]	6	Retrocorneal membrane	0.75 mg/0.15 ml	1	Yes	12	No
**Avg**	**68.1**			**15.6 ± 19.4**			**1.9 ± 0.7**		**10.6 ± 12.7**	
**5-FU + membranectomy**										
1	26	F	GDD explant [8]	0.25	Retrocorneal membrane	0.3 mg/0.1 ml	1	-	15	No
2	52	F	Trabeculectomy [11]	10	Retrocorneal membrane	N/A	3	-	7	No
3	72	M	ECCE + IOL [22]	6	Retrocorneal + angle membrane	1 mg/0.1 ml	2	-	42	No
4	65	F	DSAEK [18]	32	Retrocorneal membrane	0.5 mg/0.1 ml	2		8	Yes + DSAEK at 8 m
5	71	M	Phaco + IOL [23]	12	Retrocorneal + iris membrane	0.5 mg/0.2 ml	1	-	9	Yes + DSAEK at 6 m
**Avg**	**57.2**			**12.1 ± 12.0**			1**.8 ± 0.8**		**16.2 ± 14.8**	
**Methotrexate**										
1	40	M	DSAEK [24]	3	Retrocorneal membrane	I/C 400 mg/0.1 ml	11	Yes	11	No
2	64	M	Glaucoma + RK [25]	N/A	Retrocorneal membrane	I/V 400 mg/0.1 ml	6	N/A	N/A	No
3	67	M	Cataract + IOL [14]	6	Iris membrane	I/V 400 mg/0.1 ml	12	-	14	No
**Avg**	**57.0**			**4.5**			**9.7 ± 3.2**		**12.5 ± 2.1**	
**Mitomycin-C**										
1	60	F	Cataract + IOL [15]	84	Cystic ED on iris obstructing pupil	0.0002 mg/mL	1	-	12	No

† DSAEK: Descemet’s stripping automated endothelial keratoplasty; PKP: penetrating keratoplasty; IOL: intraocular lens; GDD: glaucoma drainage device; ECCE: extracapsular cataract extraction; AGV: Ahmed glaucoma valve; RK: radial keratotomy; I/C: intracameral; I/V: intravitreal; N/A: Not available

Our patient presented with an early, non-cystic form of ED on the iris surface without retrocorneal involvement. Among the reported cases in the literature, only two did not have corneal involvement. [14, 15] One had a recurrent membrane on the iris and the other had a cystic epithelial downgrowth on the iris surface, and they were treated with methotrexate and MMC respectively.

Among the different pharmacotherapies for ED, 5-FU had the greatest number of reported cases. The overall rate of corneal decompensation following any intraocular chemotherapy was the highest in 5FU groups in which 8 of 17 eyes (47.1%) treated with or without membranectomy developed corneal decompensation at a mean follow-up period of 12.2 ± 13.1 months. (Table 1). Combined 5-FU and membranectomy offers the most favorable outcome in terms of disease control than 5-FU alone. MTX, either intracameral or intravitreal, had a shorter injection interval and an overall greater number of injections, thus it may be considered as a second-line agent when 5-FU had failed. As for MMC, its role in non-cystic form of ED has not yet been determined.

## Data Availability

No datasets were generated or analysed during the current study.
